# Psychometric properties of the Feeling of Unsafety Scale—Arabic in general population adults

**DOI:** 10.3389/fpubh.2025.1491691

**Published:** 2025-03-28

**Authors:** Sahar Obeid, Zeinab Bitar, Diana Malaeb, Fouad Sakr, Mariam Dabbous, Souheil Hallit, Feten Fekih-Romdhane

**Affiliations:** ^1^Department of Psychology and Education, School of Arts and Sciences, Lebanese American University, Jbeil, Lebanon; ^2^Univ Rennes, Inserm, EHESP, Irset (Institut de Recherche en Santé, Environnement et Travail), UMR_S 1085, F-35000, Rennes, France; ^3^College of Pharmacy, Gulf Medical University, Ajman, United Arab Emirates; ^4^School of Pharmacy, Lebanese International University, Beirut, Lebanon; ^5^School of Medicine and Medical Sciences, Holy Spirit University of Kaslik, Jounieh, Lebanon; ^6^Department of Psychology, College of Humanities, Effat University, Jeddah, Saudi Arabia; ^7^Applied Science Research Center, Applied Science Private University, Amman, Jordan; ^8^The Tunisian Center of Early Intervention in Psychosis, Department of Psychiatry “Ibn Omrane”, Razi Hospital, Manouba, Tunisia; ^9^Faculty of Medicine of Tunis, Tunis El Manar University, Tunis, Tunisia

**Keywords:** Feeling of Unsafety Scale, Arabic, psychometric properties, validation, Lebanon

## Abstract

**Background:**

Feelings of unsafety, including fear of crime, uncertainty, or insecurity, can negatively impact individuals by reducing psychological well-being and worsening health. Validating a simple and cost-effective tool to assess the general feeling of unsafety in the Arabic-speaking population, primarily residing in the Middle-East and North-Africa (MENA) region where safety can be a major concern, would be highly beneficial. The study aimed to translate the Feeling of Unsafety Scale into Arabic (FUSA) and evaluate its psychometric properties, including internal reliability, sex invariance, composite reliability, and correlation with a measure of intolerance of uncertainty.

**Methods:**

A total of 484 Arabic-speaking adults was recruited between March and April 2024. A self-administered anonymous survey was distributed through social media using a Google Forms link. We used the FACTOR software to conduct the exploratory factor analysis (EFA) of the FUSA scale and RStudio for the confirmatory factor analysis (CFA).

**Results:**

The confirmatory factor analysis of the unidimensional model was poor; the EFA conducted on the first split subsample showed a two-factor solution, with the CFA conducted on the second split subsample showing good fit. The latter model fit indices improved even more after adding a correlation between items 2–5 due to high modification indices. The reliability of the scale was excellent as shown by the McDonald’s omega and Cronbach’s alpha values for the total score (*ω* = 0.89 and *α* = 0.90), Factor 1 = Feeling of outdoor unsafety (ω = 0.91 and α = 0.91) and Factor 2 = Feeling of indoor unsafety (ω = 0.83 and α = 0.83). Invariance was established between males and females. Good concurrent validity was attested by positive correlations between FUSA scores and intolerance of uncertainty dimensions.

**Conclusion:**

The FUSA was found to be reliable, valid, and cost-effective for measuring the general feeling of unsafety in the general population. To evaluate its practical effectiveness and further enhance data on its construct validity, future studies should assess the scale in diverse contexts and among specific populations.

## Introduction

Over the past five decades, the term “insecurity” has become increasingly common in everyday language, expanding beyond its specialized use in areas like technology and economics to become a regular feature in mass media. It has also evolved into a key concept in sociology and a central topic in political discourse (1). Meanwhile, numerous studies have explored insecurity from a psychosocial perspective, viewing it as a dynamic yet persistent state shaped by a combination of perceptions, evaluations, emotions, and concerns that emerge in the interaction between individuals and their material, social, and symbolic environments (2–4). In fact, insecurity appears to have become one of the most pressing issues of our time (5). It affects individuals both due to its psychological impacts (such as anxiety, distrust, disempowerment, and dissatisfaction) and the behaviors it triggers (e.g., reducing social activities, limiting personal freedom, relocating to safer areas, installing burglar alarms) (6–8). In addition, insecurity plays a significant role in collective life. It is influenced, at least in part, by the economic, social, and political conditions of communities, and can, in turn, impact social cohesion, fostering exclusion or delegitimization of outgroups. This dynamic may even contribute to the development of a “security ideology,” transforming a legitimate desire for safe communities into a justification for violent, racist, or xenophobic behavior (9, 10).

Feeling safe is a key issue in ensuring people’s independence, social participation and social inclusion (11). However, a large number of adults report distressing levels of feelings of unsafety (12), which negatively affect their life satisfaction (13).

### Causes of the feeling of unsafety

Safety is not just a critical part of our lives; it is essential for societies to thrive and grow (14, 15). Studies have often considered feelings of unsafety as a consequence of people’s risk perception, directly related to obvious safety-related issues (e.g., crime, terrorism, and so on) (16). Feelings of unsafety among adults can stem from various sources, including personal experiences with crime or violence, exposure to media reports on criminal activities, and societal instability. Research suggests that individuals who have experienced trauma or victimization may be more likely to perceive their environment as unsafe (17). Furthermore, socio-economic factors such as poverty and inequality can contribute to feelings of insecurity, such as living in high-crime neighborhoods (18). Additionally, political instability and widespread fear of terrorism or violence in the media can amplify perceptions of insecurity (18).

A glance at the news reveals that safety is a hot topic in today’s world, with mass shootings, racial issues, and international conflicts shaping our sense of security. Additionally, persistent local and national incidents—like terrorist attacks, police brutality, and mass shootings—constantly disrupt our daily sense of safety (19). The multifaceted nature of perceived unsafety feelings makes it difficult to be accurately measured, as it intersects with various life aspects, including health (20), work (21), and living conditions (22).

More particularly, factors that influence perceptions of safety in Lebanon include social trust, the presence of community networks, political stability, and the historical context of conflict (23–25). Additionally, cultural norms around family and neighborhood solidarity, as well as media portrayals of security threats, play significant roles in shaping individuals’ perceptions of safety in this setting (26).

### Consequences of feeling of unsafety

The feeling of insecurity is often operationalized in relation to psychological responses to crime. Rountree (27, 28) distinguishes between two key dimensions of these reactions: a cognitive dimension, which involves the perceived risk of victimization, and an affective dimension, which encompasses fear of crime. Winkel (29) further emphasizes the importance of differentiating between the subjective perception of victimization risk and the perceived negative consequences that would result from such victimization. Furstenberg (30) and Roché (31) identified two main psychological reactions to crime: fear of crime, which involves anxiety about personal safety or property, and concern about crime as a social problem, which relates to broader community security. While fear of crime is linked to personal, localized experiences, concern about crime concerns anxiety about crime affecting society as a whole and is less correlated with fear of crime.

Feelings of unsafety, including fear of crime, uncertainty, or insecurity, can negatively impact individuals by reducing psychological well-being (32) and worsening health (33). These feelings also affect social outcomes, leading to decreased social participation and lifelong learning, as people engage in more precautionary behaviors and avoid going out in the evening (34, 35). Additionally, feelings of unsafety and fear of crime in one’s neighborhood have been shown to influence mental and physical health, including depressive symptoms (36), intolerance of uncertainty (37), homicidal ideations, suicide attempts (38–40), and physical well-being indices (41). In addition, a lack of perceived safety can result in lower levels of outdoor physical activity (41), which in turn negatively impacts cardiometabolic health (42), and increases the levels of obesity (43, 44). As for the academic settings, when students feel unsafe, their academic performance tends to decline (45–47). These detrimental consequences of feeling of unsafety underscores the necessity of robust measures that enable to comprehensively assess the construct, especially in most vulnerable populations.

### Commonly used scales assessing feeling of unsafety

Most previous research has employed a range of instruments to measure unsafety feeling. Some studies have used a single-item measure [e.g., “I feel safe walking alone late at night” or “I generally feel safe”; (46, 48–52)]. However, because feeling unsafe can be influenced by a variety of both external (e.g., prevalence of crime, neighborhood walkability) and internal (e.g., anxiety, confidence) factors, and since unsafety can manifest in numerous cognitive, emotional and behavioral manifestations, relying on a single item may not capture the multifaceted nature of this experience and can significantly reduce its external validity (53). Other studies tend to measure fear of crime rather than perceived feelings of unsafety. These studies ask participants to express their fear and perceived vulnerability concerning various types of crimes (54–56). While fear of crime is a crucial aspect of unsafety feelings, it is not the only one that matters.

Another commonly used measure in scientific literature to assess feeling of unsafety is neighborhood walkability (57–61). The most widely established measure of walkability is the Neighborhood Environment Walkability Scale [NEWS; (62)], which assesses participants’ perceptions of their neighborhood design and features related to physical activity, street connectivity, accessibility of walking/cycling, neighborhood aesthetics, neighborhood satisfaction, and traffic/crime safety. An abbreviated version of this measure is also used in the literature (63). Although these measures include some questions on perceived feeling of unsafety, they do not encompass the complexity of unsafety due to their focus on environmental characteristics and neighborhood perceptions. Since unsafety depends on both the individual perceiver and the environmental conditions, a person might feel safe in some places or generally in their life but perceive their neighborhood as not being walkable (63).

To accurately measure perceived safety, it is essential to consider both subjective factors (such as perceived safety and confidence in one’s safety habits) and more objective factors (such as fear of crime and neighborhood walkability). Therefore, an effective measure of perceived unsafety should collectively focus on all these factors, rather than measuring safety with a single item or any of these factors individually.

### Feeling of Unsafety Scale-Arabic (FUSA)

Numerous studies suggest that the feeling of unsafety is multifaceted, encompassing various dimensions such as personal fear versus altruistic fear (64), fear of personal crime versus fear of property crime (12, 65), fear versus trust (66), objective risk versus subjective risk (67), cognitive perception versus affective experience versus behavior, and indoor versus outdoor feeling of unsafety (68).

To better reflect the multifaceted nature of general feeling of unsafety, Elchardus et al. (69) developed a brief 8-item self-report questionnaire that measures the construct within the general population. This questionnaire is frequently used in both policy and academic research. It contains items derived from conversations and writings about unsafety; the scale includes items that reference different aspects of time, place, specific situations, as well as feelings, behavior, and cognitive evaluation (70). Elchardus et al. (69) (in Dutch) examined the psychometric properties of the scale among adults in two samples from Flanders (Belgium). Confirmatory factor analyses support a one-factor model with good fit measures (sample 1: AGFI = 0.998 / sample 2: AGFI = 0.997), with factor loadings ranging from 0.50 to 0.75. Subsequently, an adapted version was developed after removal of two items to be also suitable for use among older adults (70). For example, the item “Out of fear that I will get mugged, I lock my car door immediately when I get in” was deemed irrelevant as older individuals, particularly the oldest age groups, tend to drive less frequently, leading to a higher likelihood of missing responses. Consequently, De Donder et al. developed an adapted version of the questionnaire, replacing the age-specific questions with two other items (70). In this context, factor analyses on data from the Belgian Ageing Studies (*N* = 39,846) provide evidence of good reliability and validity of the Elders Feelings of Unsafety (EFU) scale (70). This adapted 8-item version was chosen to be validated in the Arabic language for two main reasons. The first reason is that we are aiming to provide a measure of feeling of unsafety that can be useful among adults of all ages, including older adults. The second reason is that not all adults in Lebanon or Arab countries drive or own cars.

### The present study

Over the past decade, Arab people in general and Lebanese people in particular have endured a series of extremely distressing events, including the 2006 Lebanon War, the COVID-19 pandemic that severely impacted the year 2020, the catastrophic explosion at Beirut’s port - one of the largest non-nuclear explosions in history, and the ongoing economic crisis threatening basic human needs (71). These events have all been linked directly or indirectly to increased feeling of unsafety (71). Moreover, these political, social and economic crises have caused major challenges in the Lebanese population, leading to a worrisome increase in different types of crime, violence, and abuse, including physical assault, verbal harassment, psychological trauma, and sexual exploitation (72). Hence, it has been showed that economic deprivation creates a sense of unsafety and instability, which is likely to increase vulnerability and fear (73). Additionally, Lebanon’s governance is marked by elite dominance, leading to limited opposition and a lenient stance towards organized crime (74). The economic decline and political turmoil have further impaired the country’s ability to effectively tackle organized crime. This overall governance environment has created conditions that are conducive to organized crime, as criminal networks exploit the system’s weaknesses (74). Thus, the country would be an appropriate context to validate the Feeling of Unsafety Scale in Arabic (FUSA). Thus, this study aims to examine the psychometric properties of the FUSA in terms of factor structure, validity, reliability and measurement invariance across sex. We hypothesized that the FUSA would (1) replicate the original one-factor structure, (2) demonstrate good composite reliability and measurement invariance between sexes (males vs. females), and (3) show appropriate correlations with intolerance of uncertainty. We made sure to follow the COSMIN checklist in our paper (75); since we are adapting an existing instrument to the Arabic language, we organized the study according to the most relevant COSMIN properties:

1. Content validity: we ensured that the Arabic version captures the intended construct and is culturally appropriate by discussion the translated items with a group of experts.
2. Cross-cultural validity was ensured by conducting the confirmatory factor analysis (and exploratory factor analysis if needed).
3. Reliability by calculating the Cronbach’s alpha and McDonald’s omega values.
4. Construct validity by comparing the FUSA scores with another validated scale in Arabic (Intolerance of uncertainty).

## Methods

### Study design

Data for this cross-sectional study in Lebanon was collected via a Google Form link between March and April 2024. The project was disseminated on social media platforms (Facebook, Instagram and WhatsApp), using posters and digital advertisements that outlined key information such as the study’s purpose, eligibility criteria, benefits of participation, and contact details. The research team actively engaged with potential participants, inviting them to take part in the survey. To encourage participation in a research study, the research team typically employed strategies such as clearly communicating the study’s purpose and benefits, ensuring confidentiality and anonymity, and highlighting the potential impact of the research. Participants who agreed to participate were encouraged to share the survey link with others, using the snowball sampling technique. Given the focus of the study on perceptions of unsafety, a population-based probability sampling approach was challenging. We chose the snowball sampling technique to encourage participants who may be reluctant to engage in such studies due to the sensitive nature of the topic. To be eligible for participation, individuals had to be Lebanese residents and adult citizens (aged 18 years and above). Upon providing digital informed consent, participants were asked to complete various assessment tools in a predetermined sequence to mitigate any potential order-related biases. It is important to emphasize that the survey was anonymous, and participants voluntarily completed it without receiving any compensation.

### Minimal sample size calculation

A minimum of 10 (76) and 3–20 (77) participants per scale’s item was needed for the exploratory and confirmatory factor analyses, respectively.

### Translation procedure

Following Beaton’s guidelines (78), the forward-backward translation method was used for the scale. Initially, two Lebanese translators, who were not involved in the study, translated the English version into Arabic. Then, two Lebanese psychologists fluent in English back-translated the Arabic version into English. To verify the accuracy of the translation and ensure face and content validity, the original English version was compared with the translated version. Any discrepancies were identified and resolved by a committee consisting of the research team and the translators (79). Additionally, the scale was adapted to the Arab context to ensure that item wording was clearly understood and that there was conceptual equivalence between the original and Arabic versions in both contexts (80). After translation and adaptation, a pilot study with 30 participants was conducted to confirm that all questions were comprehensible; no changes were made following the pilot study.

### Questionnaire

The survey, taking approximately 5 min to be filled, was developed in Arabic, the official language of Lebanon, and structured into three main sections. The initial section served as an online consent checkpoint, ensuring voluntary participation and addressing ethical concerns including confidentiality and anonymity of responses. It also provided an overview of the project and instructions for completing the questionnaire. The second part of the survey aimed to collect socio-demographic data from participants, including details such as age, sex, and Household Crowding Index (HCI). The latter reflects the Socioeconomic Status (SES) of the individual and is calculated by dividing the number of persons by the number of rooms in the house except the kitchen and bathrooms (81); higher HCI scores reflect a lower SES. The third section encompassed two detailed measures, as outlined below.

#### The Feeling of Unsafety Scale – Arabic

The Feeling of Unsafety Scale – Arabic (FUSA) adapted from the *Elders Feelings of Unsafety (EFU)* scale contains eight items based on a 5-point scale, ranging from 1 (feeling completely safe) to 5 (feeling completely unsafe) (e.g., “You have to be extra careful when you are out on the streets at night”) (17). Higher scores indicate higher feeling of unsafety. Permission for translation and validation of the scale was obtained from Pr. Liesbeth De Donder.

#### The intolerance of uncertainty scale

The Intolerance of Uncertainty Scale (IUS-12) (82), validated in Lebanon (83), a shortened version of the IUS-27, consists of 12 items scored on a Likert scale from 1 (Not at all characteristic of me) to 5 (Very characteristic of me). The IUS-12 includes two subscales: prospective anxiety and inhibitory anxiety, with higher scores indicating higher levels of anxiety. The reliability coefficients were excellent for the prospective anxiety (*ω* = 0.91 and *α* = 0.91) and inhibitory anxiety (ω = 0.92 and α = 0.91) subscales. Noting that the Arabic validation showed a bi-dimensional model with an excellent internal reliability for the prospective anxiety (ω = 0.85 / α = 0.85) and inhibitory anxiety (ω = 0.87 / α = 0.87) (83).

### Statistical analysis

The factor structure of the FUSA was explored using the EFA and CFA using RStudio (Version 4.2.2.). First, we checked the original items from the unidimensional version, revealing a very bad fit to the present data. Consequently, we decided to divide the sample into two subsamples of 33% (*n* = 167) and 67% (*n* = 317) randomly using the SPSS software “select cases” option. EFA was performed on the first subsample (*n* = 167) based on the principal component extraction approach and the oblimin rotation. Factors were extracted for further analysis based on eigenvalues on the Scree plot (Factors with eigenvalues >1). The data viability for factorability was evaluated through Kaiser–Meyer–Olkin (KMO) measure of sample adequacy (Values >0.7) and Bartlett’s test of Sphericity (*p* < 0.05) (84).

After that, we performed a CFA on the other subsample (*n* = 317) to confirm the derived factor structure from EFA. A confirmatory factor analysis was conducted using the “Lavaan” and “SemTools” package (85, 86). Values greater than 0.90 for the CFI and TLI, values closer to 1.00 for the GFI indicate good model fit. However, values for the RMSEA are expected to be at or below 0.08 to represent a good model fit, whereas values ≤0.10 are indicative of an acceptable fit (87, 88). Correlations were added between items’ residuals if the modification indices were high.

To examine sex invariance of the FUSA scores, we conducted multi-group CFA assessed at the configural, metric, and scalar levels. Following the recommendations of Chen (89), we accepted ΔCFI ≤ 0.010 and ΔRMSEA ≤ 0.015 or ΔSRMR ≤ 0.010 as evidence of invariance.

The FUSA score was considered normally distributed as shown by values of the skewness (= −0.772) and kurtosis (= 1.286). Pearson correlation test was used to correlate two scores, whereas the independent t test was used to compare the mean feeling of unsafety scores between males and females. Cronbach’s alpha and McDonald’s omega were computed to assess the reliability of the scales used.

## Results

Four hundred eighty-four adults filled the survey, with a mean age of 27.74 ± 11.17 years, 68.4% females and a mean HCI of 1.12 ± 0.47 person/room.

First, the unidimensional version, revealed a poor fit: RMSEA of 0.20 [90% CI 0.18, 0.22], CFI of 0.83, TLI of 0.77, a GFI of 0.80 and an SRMR of 0.10.

EFA with oblimin rotation extracted two factors from the FUSA scale. The proportion variance was 65%, with Factor 1 (feeling of outdoor safety) and Factor 2 (feeling of indoor safety) explaining 38 and 27% of the variance, respectively, (KMO = 0.86 and *p* value for the Bartlett’s test of sphericity <0.001). Table 1 shows the factor loadings of 8 items of the FUSA scale for two extracted factors from the EFA.

**Table 1:** Factor loadings of 8 items of the FUSA scale extracted from the exploratory factor analysis.

		Extracted factors^*^	
Items		F1: Feeling of outdoor safety	F2: Feeling of indoor safety
1	You have to be extra careful when you are out on the streets at night.	**0.94**	−0.03
2	These days, it is not safe to be out on the streets at night.	**0.90**	−0.04
3	These last 10 years, the streets have become less safe.	**0.70**	0.14
4	After nightfall, I do not open the door when someone rings.	−0.10	**0.85**
5	These days, it is not safe to let children out on the streets without supervision.	**0.78**	0.04
6	I seldom go out alone because I am afraid of being mugged.	0.08	**0.78**
7	These days an alarm system is more than just a gadget.	0.32	**0.49**
8	When I go away on holiday, I do not dare to leave my house unwatched.	0.05	**0.68**

^*^Exploratory factor analysis with oblimin rotation; numbers in bold are retained on the specific factor.

A confirmatory factor analysis was conducted using the two-factor solution that derived from the EFA; the results showed good fit: CFI of 0.93, TLI of 0.89, a GFI of 0.91 and an SRMR of 0.05, but a poor RMSEA of 0.14 [90% CI 0.12, 0.16]. To improve this main model, we examined the Modification Index (MI). The MI showed a strong positive covariance between item 2 and 5. The new modified model showed good fit model indices, a CFI of 0.95, a TLI of 0.92, a GFI of 0.93, an SRMR of 0.05 and a decreased RMSEA of 0.12 [90% CI of RMSEA (0.10, 0.14)].

After reviewing the modification indices, we investigated this issue further and performed the second-order CFA following the same method as done on the two-factor model. The fit indices of the second order CFA were acceptable as follows: CFI =0.958, TLI = 0.902, SRMR = 0.048 and RMSEA of 0.130 [90% CI of RMSEA (0.108, 0.153)]. We believe that the adjustment involving the second-order CFA model solved this issue by showing improved fit and no negative variances.

**Figure 1:**
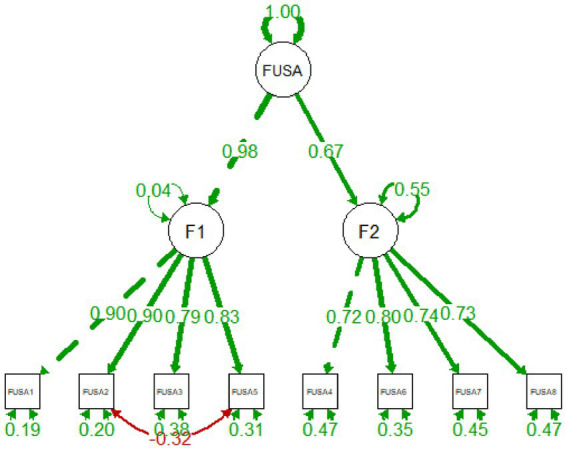
Standardized loading factors of the Feeling of Unsafety Scale in Arabic derived from the second-order CFA model are presented in green. Covariance between FUSA2 and FUSA5 are presented in red to highlight the strength and significance of their relationship. Loadings are standardized to the FUSA factor.

It is of note that the reliability of the scale was excellent as shown by the McDonald’s omega and Cronbach’s alpha values for the total score (*ω* = 0.89 and *α* = 0.90), Factor 1 = Feeling of outdoor unsafety (ω = 0.91 and α = 0.91) and Factor 2 = Feeling of indoor unsafety (ω = 0.83 and α = 0.83). The standardized loading factors deriving from the second-order CFA model are summarized in Figure 1.

### Sex invariance (using the total sample)

We were able to show invariance across sex at the configural, metric, and scalar levels (Table 2). A significantly higher mean feeling of unsafety total score (30.62 ± 6.12 vs. 28.97 ± 5.68; *t* (482) = −2.82; *p* = 0.005) and Factor 1 = Feeling of outdoor unsafety (16.34 ± 3.36 vs. 15.33 ± 3.08; *t* (482) = −3.13; *p* = 0.002), but not Factor 2 = Feeling of indoor unsafety (14.28 ± 3.48 vs. 13.63 ± 3.23; *t* (482) = −1.95; *p* = 0.052), were found in females vs. males.

**Table 2:** Measurement invariance of the Feeling of Unsafety Scale – Arabic (FUSA) scale across sex in the total sample.

Model	CFI	RMSEA	SRMR	Model comparison	ΔCFI	ΔRMSEA	ΔSRMR
Configural	0.947	0.110	0.077	Configural and metric	−0.005	0.11	0.011
Metric	0.952	0.110	0.067	Metric and scalar	−0.003	0.06	0.011
Scalar	0.949	0.104	0.077				

### Concurrent validity

Higher feeling of unsafety was significantly associated with higher prospective anxiety (r = 0.21, *p* = 0.001) and inhibitory anxiety (r = 0.18; *p* < 0.001) (see Table 3).

**Table 3:** Pearson correlation matrix between continuous scores.

	1	2	3	4
1. FUSA–Total scores	1			
2. FUSA–Feeling of outdoor unsafety	0.89^***^	1		
3. FUSA–Feeling of indoor unsafety	0.90^***^	0.61^***^	1	
4. Intolerance of Uncertainty–Prospective anxiety dimension	0.21^***^	0.27^***^	0.11^*^	1
5. Intolerance of Uncertainty–Inhibitory anxiety dimension	0.18^***^	0.16^***^	0.17^***^	0.78^***^

FUSA, Feelings of Unsafety Scale – Arabic. *p < 0.05; ***p < 0.001.

## Discussion

The findings from this study suggest that the FUSA scale can reliably and validly measure general feelings of unsafety in the Arabic-speaking adult general population. In addition, the results support the measurement invariance of the Arabic scale across sex group. Overall, the FUSA is suggested to be a brief, easy-to-use and psychometrically sound measure of unsafety feelings that is suitable for use in the Arab contexts.

In our study, the instrument exhibited a two-factor structure, contrary to the one-factor model presented in the original paper on adults living in Flanders (Belgium) (69) and the validation conducted among Belgian older adults (17). Factor 1 includes items 1, 2, 3 and 5 (e.g., “These days, it is not safe to be out on the streets at night”), was labelled “Feeling of outdoor unsafety,” and reflects feeling unsafe while being on the streets. Factor 2 includes items 4, 6, 7, and 8 (e.g., “After nightfall, I do not open the door when someone rings”), was labelled “Feeling of indoor unsafety,” and reflects unsafety feelings related to being at home. Our findings thus support distinguishing between two different dimensions, rather than relying solely on one dimension or the total FUSA score. Dividing items into two factors about feeling unsafe and while including references to specific spatial contexts can be highly relevant in assessing perceived safety issues accurately. Consequently, it can be suggested that the two-factor model is more effective in capturing the various dimensions of the feeling of unsafety (64). However, future studies are still required to confirm whether this two-factor solution holds up across different countries, cultural groups, populations and settings. Despite the brevity of FUSA, its reliability was remarkably high. In our study, the internal reliability for the total score ranged from acceptable to excellent (*ω* = 0.89 and *α* = 0.90), closely mirroring the results from the Belgian Ageing Studies (*N* = 39,846), which demonstrated good reliability with an acceptable Cronbach’s alpha of 0.89 (70).

For item 7, we decided to retain it in Factor 2 rather than remove it, as the item would be deleted if the cross-loadings different by less than 0.15 from the item’s highest factor loading (90), which is not the case here. We added a correlation between residuals of items 2 and 5 since the modification indices were high; the reason behind it is that both items are related to the general perception of safety in public spaces, with one related to adults’ safety and the other to children’s safety. Usually, the two items should be positively correlated since a person who feels unsafe on the streets at night might also perceive children being unsafe too in similar situations. Since the negative correlation between items are negative, we hypothesize that individuals with a higher levels of safety concerns may not share the same perception about children; a person thinks that adults can protect themselves in case of danger, while this does not apply to children who are more vulnerable and require supervision.

Another finding of our research is that measurement invariance was supported across sex groups. Therefore, the FUSA can be used to make psychometrically sound comparisons between individuals with different characteristics (males versus females). It is important to note that previous papers did not assess this important psychometric property, hence, the originality of our paper. Future studies are recommended to analyze in depth the measurement invariance before making any conclusions about sex differences in terms of feeling of unsafety. A significantly higher mean of feeling of unsafety was found in females compared to males, which is in line with previous studies (91, 92). More precisely, higher feeling of outdoor unsafety was found in females compared to males reflecting the integrated nature of the home and neighborhood as a psychosocial environment affecting the feeling of unsafety (93). Differences between outdoor and indoor feeling of unsafety between males and females can be explained by the exposure to intimate partner violence (94–96), perceived threat of rape, patriarchy, differential socialization, and physical vulnerability (97, 98). A lower level of perceived safety among females is not just a random local phenomenon but a multicultural one (99). Our findings align with those of May et al. (100) who found that females experience significantly higher feelings of unsafety and fear of crime victimization and are more likely to perceive themselves at greater risk of victimization than men, specifically in outdoor areas where they have less control and power. Additionally, females engage in avoidance behavior, more often than males, such as avoiding certain outdoor activities or places due to fear of violence or crime victimization such as woods, nightlife activities, and outdoor activities (101). The fact that females fear violent crimes more than males, despite being less frequently victimized, is known as the “paradox of fear of victimization” [e.g., (67, 102)]. The disparity in perceived safety between sexes is also based on the assumption that females are considered more vulnerable to criminal acts, not only due to the likelihood of victimization but also because of their inability to manage the consequences of physical, psychological, and economic losses resulting from their victimization (103, 104). According to Rader et al. (105) and Scott (106), in the context of the social learning theory of the socialization of sex perception of safety by Rader and Hayes (107), females are exposed to information indicating that they are physically vulnerable to victimization; that there is a higher likelihood of victimization by male attackers, and that they are more likely to be victimized in public places at night.

Lastly and as expected, our findings revealed that higher feeling of unsafety was significantly associated with higher intolerance to uncertainty, which provides support for the concurrent validity of the FUSA. In line with our findings, the concept of intolerance of uncertainty has recently been theorized as “a felt sense of unsafety,” and people who are dispositionally more intolerant to uncertainty about the presence of safety tend to exhibit more feelings of unsafety in life situations (for review, see (108)). When people judge a situation or environment to be especially unsafe, when they feel they have little personal control over its occurrence (109), and when people are uncertain about the presence of safety, the stress response is released (108). Consequently, when unsafety feeling or victimization is psychologically proximate, then uncertainty and the lack of cognitive closure will bring with it a sense of negative affect that makes worry more frequent (37).

### Clinical implications

This study has significant implications, providing a valid and reliable tool for clinicians and researchers who work in Arab settings to assess feelings of unsafety among the general population, thereby enhancing research opportunities in Arabic-speaking populations, especially those who live in the increasingly unsafe MENA region. Moreover, the validation of the FUSA may help understand the unsafety feeling, enable the development of strategies to reduce social distrust and promote healthy social coexistence and welfare. Additionally, the availability of an Arabic version of the FUSA facilitates cross-cultural comparisons.

### Limitations

This study has several limitations similar to most research. The sample was obtained through snowball sampling technique, which may limit the generalizability of the findings. Additionally, the cross-sectional design of this study prevents us from determining causality or the temporal sequence of relationships between variables. Furthermore, some important psychometric properties, such as inter-rater and test–retest reliability, were not examined in this study. Responses were self-reported by participants, which predisposes us to an information bias. To address these limitations, future research should include more representative samples of Lebanese adults (in clinical and non-clinical settings), including minorities and individuals with different sexual orientations, and adopt longitudinal and cross-cultural approaches. Further studies are also needed to explore whether the FUSA scale can be applied to Arabic-speaking adults from other Arab countries with different social and cultural backgrounds. A further limitation of the study could be that the use of the Intolerance of Uncertainty Scale to assess convergent validity may not fully capture the situational nature of fear responses, as the scale primarily measures a personal trait rather than a specific fear-related reaction.

Moreover, it is important to acknowledge that the depth and fluidity of the concept of feeling of unsafety, as described by Amerio and Roccato (110), might be underrepresented in such a simplified scale. Insecurity, as a multifaceted phenomenon encompassing perceptions, emotions, and social contexts, may require a more nuanced approach to measurement in order to fully capture its complexity and the range of factors influencing individuals’ sense of safety or insecurity. In addition, while fear of crime often focuses on personal anxiety related to criminal activity, unsafety encompasses broader social and physical dynamics, including the breakdown of social ties and visible disorder, which can exacerbate feelings of insecurity. In this sense, a more comprehensive approach would integrate these aspects to better capture the multifaceted nature of unsafety. We may need to reconsider the scope and content of the FUSA to reflect this wider understanding, moving beyond fear of crime to address the broader structural factors that contribute to unsafety. Lastly, readers and the broader scientific community should bear in mind that the FUSA provides a broad measure of unsafety or fear of crime without delving into its specific sources, such as social cohesion or fear of crime. Finally, CFA was used to test the hypothesis that the fear of unsafety construct is manifestation of a single factor. While this analysis is widely accepted in scale validation research, other approaches, such as Rasch Analysis can be valuable for assessing psychometric properties of ordinal scales and should be considered in future validation studies of the FUSA.

## Conclusion

Our findings provide new evidence on the good psychometric properties of the FUSA, supporting its suitability for assessing the general feeling of unsafety among Arabic-speaking adults, particularly in Lebanon. In particular, the FUSA was found to be reliable, valid, and cost-effective for measuring the general feeling of unsafety in the general population. However, further research is recommended to extend the validation of this translated measure to a more diverse Arabic-speaking population. This expansion would enhance the generalizability and applicability of the FUSA across various cultural contexts within the Arabic-speaking community. Lastly, we acknowledge the importance of assessing the stability of the Unsafety construct, and future research could explore its temporal consistency to better understand its role in behavior over time.

## Data Availability

The raw data supporting the conclusions of this article will be made available by the authors, without undue reservation.
